# Loss of ATRX confers DNA repair defects and PARP inhibitor sensitivity

**DOI:** 10.1016/j.tranon.2021.101147

**Published:** 2021-06-09

**Authors:** Jennifer Garbarino, Jillian Eckroate, Ranjini K. Sundaram, Ryan B. Jensen, Ranjit S. Bindra

**Affiliations:** a Department of Molecular Biochemistry and Biophysics, Yale University, New Haven, CT 06511, USA; b Department of Therapeutic Radiology, Yale University School of Medicine, New Haven, CT 06511, USA

**Keywords:** ATRX, PARP inhibitor, IDH1 R132H, Glioma, DNA damage response

## Abstract

Alpha Thalassemia/Mental Retardation Syndrome X-Linked (ATRX) is mutated frequently in gliomas and represents a potential target for cancer therapies. ATRX is known to function as a histone chaperone that helps incorporate histone variant, H3.3, into the genome. Studies have implicated ATRX in key DNA damage response (DDR) pathways but a distinct role in DNA repair has yet to be fully elucidated. To further investigate the function of ATRX in the DDR, we created isogenic wild-type (WT) and ATRX knockout (KO) model cell lines using CRISPR-based gene targeting. These studies revealed that loss of ATRX confers sensitivity to poly(ADP)-ribose polymerase (PARP) inhibitors, which was linked to an increase in replication stress, as detected by increased activation of the ataxia telangiectasia and Rad3-related (ATR) signaling axis. ATRX mutations frequently co-occur with mutations in isocitrate dehydrogenase-1 and -2 (IDH1/2), and the latter mutations also induce HR defects and PARP inhibitor sensitivity. We found that the magnitude of PARP inhibitor sensitivity was equal in the context of each mutation alone, although no further sensitization was observed in combination, suggesting an epistatic interaction. Finally, we observed enhanced synergistic tumor cell killing in ATRX KO cells with ATR and PARP inhibition, which is commonly seen in HR-defective cells. Taken together, these data reveal that ATRX may be used as a molecular marker for DDR defects and PARP inhibitor sensitivity, independent of IDH1/2 mutations. These data highlight the important role of common glioma-associated mutations in the regulation of DDR, and novel avenues for molecularly guided therapeutic intervention.

## Highlights

• Drug screen shows that ATRX KO leads to PARP inhibitor sensitivity in glioma cells.
• PARPi leads to greater levels of replication stress in ATRX KO cells than WT.
• IDH1 R132H and ATRX KO have similar levels of PARP inhibitor sensitivity.
• ATRi and PARPi have greater synergy in ATRX KO cells.

## Introduction

ATRX (Alpha thalassemia retardation syndrome X-linked) has been studied extensively for its role in the syndrome it is named after, but was only recently found to have importance in cancers such as pancreatic neuroendocrine tumors (PanNet) [1] and gliomas [2]. ATRX is an ATP dependent chromatin modifier that helps to deposit histone variant 3.3 (H3.3) into the genome at heterochromatin [3], pericentromic regions [4], rDNA [5], and other structured regions. ATRX is now a diagnostic marker for gliomas due to its frequency and distinguishing characteristics, as nearly 30% of younger glioma patients have an ATRX mutation [6]. ATRX loss is also necessary but not sufficient for the Alternative-Lengthening of Telomeres pathway, which occurs in 10–15% of tumors [7]. ATRX has been implicated in a number of DNA damage response (DDR) pathways, including replication stress response [8], [9], [10], homologous recombination (HR) [11,12] and non-homologous end joining (NHEJ) [13]. However, it has yet to be determined whether loss of ATRX confers sensitivity to DDR inhibitors in a glioma model, despite its frequent occurrence.

An important pathway that can connect many of these processes is the signaling of replication stress, or damage that affects the ability of the cells to properly duplicate its DNA. This can be detected through ATR activation. Ataxia telangiectasia and Rad3‑related (ATR) is a kinase that is recruited to single strand breaks by Replication Protein A (RPA). ATR phosphorylates RPA (at S33) as well as other proteins to activate DNA repair pathways [14], [15], [16]. CHK1 is also phosphorylated (at S317 and S345) by ATR which halts the cell cycle for replication stress to be resolved as well as activating downstream, DNA repair factors [17]. Investigating the signaling in this pathway allows for better understanding for the levels of replication stress in a cell. Additionally, inhibition of this pathway has been shown to have anti-cancer effects through the use of ATR inhibitors [18,19].

ATRX loss often co-occurs with glioma-associated mutations in other genes, such as isocitrate dehydrogenase-1 and -2 (IDH1/2). IDH1/2 encode citric acid cycle enzymes which convert isocitrate into alpha-ketoglutarate (αKG), and neomorphic mutations in these genes converts alpha-ketoglutarate into the oncometabolite, 2-hydroxyglutarate (2-HG). 2HG competitively inhibits αKG-dependent dioxygenase proteins, which induces profound epigenetic alterations and impaired differentiation [20,21]. This leads to increases in patient survival as these tumors [22]. We and others recently demonstrated that 2HG induces HR defects and sensitivity to poly (ADP)-ribose polymerase (PARP) inhibitors [23], [24], [25], [26], [27]. PARP inhibitors have been found to be effective in multiple DDR deficient cancers and have been FDA approved for multiple indications such as breast and ovarian cancers [28,29].

However, it has yet to be fully elucidated how ATRX and IDH1/2 mutations interact with regard to modulation of the DDR and sensitivity to PARP inhibition. While one study reported that loss of ATRX impaired NHEJ [13], a subsequent study from the same group suggested that ATRX loss increased DDR activity specifically in the context of IDH1/2 mutations [30]. These conflicting results were derived largely from rodent models, and thus, additional data are required in human glioma models.

It is clear that further study of ATRX in the context of glioma is important to better understand its function and develop potential therapeutics. It is also necessary to identify how the DDR pathways in ATRX deficient cells are modulated when in the presence of common co-occurring mutations such as IDH1 R132H to better predict response to potential therapies. By modeling these mutations independently and in combination, further insights can be gained into the DDR pathway as well as the potential success of DDR inhibitors and DNA damaging agents selectively targeting these cells.

To further investigate the function of ATRX in the DDR, we created isogenic wild-type (WT) and ATRX knockout (KO) model cell lines using CRISPR-based gene targeting and performed a focused drug screen for novel synthetic lethal interactions with DDR inhibitors and DNA damaging agents. These studies revealed that loss of ATRX confers sensitivity to poly(ADP)-ribose polymerase (PARP) inhibitors, which was linked to an increase in replication stress, as detected by increased activation of the ataxia telangiectasia and Rad3-related (ATR) signaling axis. We found that the magnitude of PARP inhibitor sensitivity was equal in cell line models with ATR loss and IDH1 mutations alone. No further sensitization was observed in combination, suggesting an epistatic interaction. Finally, we observed enhanced synergistic tumor cell killing in ATRX KO cells with combined ATR and PARP inhibition, which is commonly seen in HR-defective cells [31], [32], [33]. Taken together, these data reveal that ATRX may be used as a molecular marker for DDR defects and PARP inhibitor sensitivity, which is independent of IDH1/2 mutations.

## Materials and methods

### Antibodies and reagents

Antibodies: ATRX: Millipore Sigma 39F MABE1798 and Santa Cruz sc-55584 were used 1:1000 overnight in 1X TBST after 5% milk block for western blot, pChk1: CST 2341 was used at 1:500 overnight at 5% BSA in 1X TBST. Olaparib, AZD6738 and other drug screen compounds were purchased through Selleckchem.

### Cell culture

Immortalized astrocytes were a gift from Tim Chan. U251 and LN229 glioma cells were also used in this manuscript. All cell lines were grown in DMEM with 10% FBS. For siRNA experiments, siATRX from Dharmacon (006524–05) was transfected into cells using Life Technologies Lipofectamine RNAiMax (13778) and imaged 96 h later. IDH1 overexpression cell lines were created using lentivirus with the plasmid pSLIK-IDH1-R132H-FLAG (Addgene plasmid # 66803).

### Clonogenic survival assay

Cells were seeded at a three-fold dilution between 9000 and 37 cells per well of a 6 well plate in triplicate and incubated in multiple drug concentrations for 14 days. Plates were washed with PBS, stained with crystal violet for 1 h, and quantified.

### CRISPR knockout and screening

To create the CRISPR knockout cell line, a guide to exon 9 (5′-AAATGCATTCTACGCAACCT-3′) was cloned into the MLM3636 plasmid (Addgene 43860). This plasmid along with a Cas9 plasmid were nucleofected into the cells and after at least 72 h, successful Cas9 cleaving was validated using a T7 endonuclease assay. Cells were then diluted to single cells into 9, 96-well plates. Wells were then screened for colonies and replica plated to 96 well plates for imaging (Greiner screenstar 655866), and further passaged. Imaging plates were stained using the immunofluorescence protocol below. Cells with diminished ATRX foci were then identified visually using the Cytation 3 (BioTek). Wells with images containing less than 10 cells were screened manually.

### Flow cytometry

Cells were seeded 48 h before experiments and harvested and fixed in 70% ethanol. RNase/Propidium Iodide (PI) buffer (BD Biosciences) was added to samples 30 min before analysis on an LSRII FACS machine (BD biosciences). Experiments performed in triplicate and analyzed using FlowJo software.

### Immunofluorescence

For immunofluorescence assays, cells were seeded in chamber slides (Millipore PEZGS0816). Cells were treated as indicated. For Cyclin A (Santa Cruz B-8 sc-271682) or ATRX (Millipore), cells were fixed in 4% PFA, 0.02% Triton in PBS for 15 min. Cells were then permeabilized/blocked for 1 h in 5% BSA, 0.5% Triton in PBS and in primary overnight at 1:500 in blocking solution. Secondary (Alexa Fluor 647) was diluted 1:1000 for 1 h. For pRPA32 S33 (Bethyl A300–246A) protocol was based on Shiotani et al. [34]. Chamber slides were imaged on a Keyence BZ-X800. Foci were analyzed using the Focinator [35].

### Sequencing

Genomic DNA was purified from cells and CRISPR region was amplified. TOPO reaction was performed using TOPO TA cloning kit (Thermo Fisher 450071) and transformed into DH5α cells. DNA was amplified through colony PCR using SapphireAmp fast PCR (Takara Bio RR350), and PCR cleanup was performed with ExoSAP-IT™ (Applied Biosystems 75001). Sequencing was performed by the Yale Keck Biotechnology Resource Laboratory.

### Short term viability assay

Cells were plated at 2000 cells per well of a 96 well plate. The following day cells were treated with various concentrations of drug as indicated. 96 h after of drug treatment, cells were washed in 1X PBS, fixed in 4% formaldehyde and stained with Hoechst at 1 µg/ml. Plates were imaged on a Cytation 3 (BioTek) and cells were counted using CellProfiler (http://cellprofiler.org/). For synergy assays, synergy was calculated using the Loewe method through Combenefit [36].

### Statistics

Student 2 tailed T test was performed to compare groups using GraphPad Prism. Asterisks indicate levels of significance/p value (*≤0.05, **≤0.01,*** ≤0.001,**** ≤0.0001). Error bars indicate standard deviation.

## Results

### ATRX screening platform to generate ATRX knockout astrocytes

**Fig. 1:**
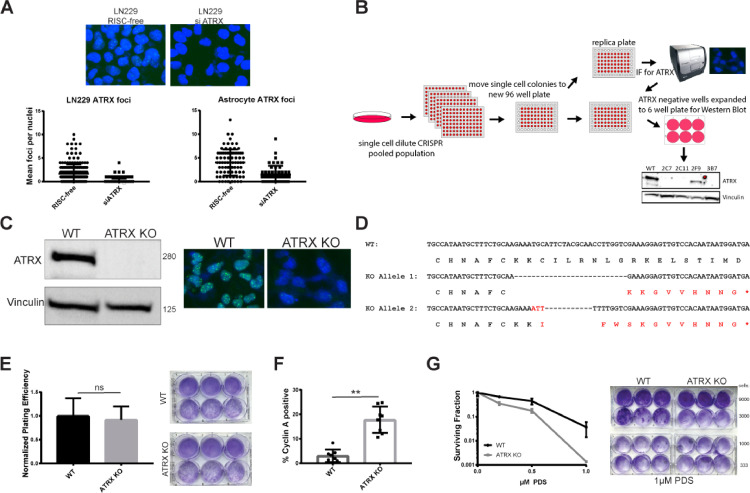
Immunofluorescence screening pipeline successful in identifying ATRX CRISPR knockout in immortalized astrocyte cells. (A) Validation of immunofluorescence ATRX foci 96 h after siRNA transfection in LN229 and immortalized astrocyte cells. (B) Schematic of immunofluorescence screening pipeline for CRISPR clones. (C) ATRX KO clone in immortalized astrocytes further validated through western blot and immunofluorescence. (D) Sequencing data showing biallelic knockout of ATRX. (E) Representative plating efficiency in parental and wildtype cell lines. Untreated cells plated and two weeks later and colonies counted. Mean ± standard deviation plotted and Student's *T* test shows no significant difference between conditions. (F) Cyclin *A* staining was performed, and positive cells were scored through immunofluorescence intensity greater than 20,000 in 16-bit images. Representative results from 6 fields of view shown as mean ± standard deviation. Student's *T* test shows significant (*P* < 0.01) (G) Sensitivity to pyridostatin after 14 day clonogenic survival assay. Cells were treated with 0.1, 0.5 and 1 µM pyridostatin. Mean ± standard deviation plotted.

High-throughput immunofluorescent microcopy can be utilized as a powerful technique to screen for loss of protein expression following CRISPR-Cas9 based knockout strategies. Many DNA repair factors are recruited to DNA damage sites and can be visualized through immunofluorescence as foci, punctate signals of multiple proteins recruited to the same location. ATRX forms foci at baseline (i.e., with no exogenously added DNA damage) and these foci were greatly diminished with siRNA to ATRX in LN229 GMB cells and immortalized human astrocytes (Fig. 1A). These findings prompted us to develop a novel imaging cytometry-based assay for ATRX baseline foci levels, which could serve as a high-throughput method to rapidly identify biallelic ATRX KO cell lines created using CRISPR-Cas9. The schema for our overall approach is shown in Fig. 1B. Guide RNAs (gRNAs) were optimized to target exon 9 of ATRX, and co-transfected with Cas9 into immortalized astrocytes, followed by limiting dilution (1 cell/well) into 96-well plates. As opposed to other methods that use a fluorescent marker for potential positivity [37,38] this method allows screening by ATRX protein levels. Using this pipeline, we found human astrocyte clones with successful ATRX KO (Supplemental Fig. 1A). To further validate one representative clone, we performed western blot and foci analysis to confirm loss of the ATRX protein and attenuated baseline ATRX foci levels, respectively (Fig. 1C). We then used DNA sequencing analysis coupled with TOPO cloning to confirm biallelic loss of ATRX (representative sequences shown Fig. 1D). Two different deletions were found in these cells, each causing a frame shift leading to a stop codon. This stop codon prevents the entire protein from being translated and can lead to nonsense mediated decay of the entire transcript [39]. The predicted truncated protein disrupts the ADD domain of ATRX [4], suggesting that any truncated protein not detected by western blot or immunofluorescence would not be functional, if at all present.

ATRX loss has been associated with cell cycle changes [8], which prompted us to further characterize the growth patterns and cell cycle phase distribution in our WT and KO models. We determined that our ATRX KO cell line grew at the same rate as the parental astrocytes as seen previously with ATRX KO in HeLa cells, and we did not detect any differences in plating efficiencies [8,11] (Fig. 1E). We found a significantly larger percentage of ATRX knockout cells were cyclin A-positive in comparison to isogenic wild-type counterparts (Fig. 1F), which suggests an increased S/G_2_ population in ATRX KO cells. To ensure that this was not an effect specific to our KO model, we also created a doxycycline (dox)-inducible shRNA to ATRX cell line in the glioma cell line U251 (Supplemental Fig. 2A). We determined that there is a similar increase in S/G2 phase in these cells, specifically in S phase through propidium iodide (PI) staining (Supplemental Fig. 2B,C). This suggests that ATRX deficiency leads increased time in S-phase and potential sensitivity to DNA damaging and/or repair inhibitors.

Finally, we found that ATRX KO cells have functional defects similar to those previously reported [38] and are particularly sensitive to pyridostatin (PDS), a *G*-quadruplex stabilizer (Fig. 1G). Reduced survival of ATRX KO cells in response to PDS treatment has been suggested to stem from a lack of ATRX H3.3 deposition at *G*-rich sites, an important step for resolution of these topologically complex DNA structures [10,38,40]. Taken together, these data confirm our ability to rapidly isolate, characterize, and functionally validate ATRX WT and KO isogenic GBM model cell line pairs.

### ATRX knockout drug screen reveals PARP inhibitor sensitivity

**Fig. 2:**
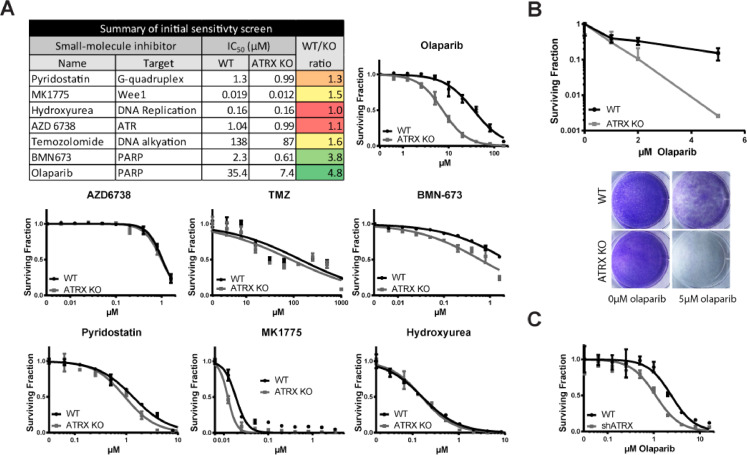
ATRX KO in immortalized astrocytes demonstrates PARP inhibitor sensitivity in a focused DNA repair drug screen. (A) Representative IC_50_ plots of short-term viability assays in immortalized astrocytes 96 h after drug treatment. (B) Sensitivity to olaparib in ATRX KO cells after 14 day clonogenic survival assay. (C) Short term viability assay and western blot show knockdown of ATRX also leads to PARPi sensitivity in U251s. shATRX induced with 1 µg/ml of doxycycline for greater than 96 h prior to experiment. For (A–C) Mean ± standard deviation plotted.

Given the previous evidence of a possible role for ATRX in the DDR, we performed a focused drug screen with unique DNA damaging agents and repair inhibitors to identify potential synthetic lethal interactions that are associated with ATRX loss. Screening was performed in the active concentration ranges for the molecules, and WT and ATRX KO cell lines were analyzed in parallel. Results of the screen are shown in Fig. 2A, along with a summary table of the calculated IC_50_ (the concentration of compound required for 50% cell kill) for each drug in the ATRX WT and KO cell lines. We included pyridostatin as a positive control as ATRX KO cells are known to be sensitive to this compound [38]. We observed a detectable differential (albeit modest) against ATRX KO versus WT cells. As anticipated, the differential survival was greater in the clonogenic survival assay (Fig. 1G), as the ability to survive for 96 h does not completely reflect the ability to form a colony of cells over two weeks. We also found that treatment with the Wee1 inhibitor, MK1775, also selectively targeted ATRX KO cells (Fig. 2A), which is consistent with previously published studies [41].

We detected a robust synthetic lethal interaction between loss of ATRX and the two PARPi's, olaparib and BMN673 (talazoparib). Notably, olaparib treatment resulted in an almost 5-fold reduction in the IC_50_ response in ATRX KO cells compared to WT. We further validated the synthetic lethal interaction by clonogenic survival assays (Fig. 2B). As shown in the representative crystal violet-stained plates, very few ATRX KO colonies survive at 5 µM olaparib, despite many colonies still present in the WT treated cells. We then validated the differential PARP inhibitor sensitivity in our U251 line containing ATRX shRNA. Upon ATRX knockdown, U251 cells show sensitivity to olaparib (Fig. 2C) and BMN-673 (Supplemental Fig. 3A). PARP inhibitor sensitivity was also further confirmed in a clonogenic survival assay with olaparib in this dox-inducible shATRX knockdown model (Supplemental Fig. 3B). Collectively, these data suggest that loss of ATRX confers significant sensitivity to PARP inhibitors.

### Olaparib leads to increased replication stress in ATRX KO cells

**Fig. 3:**
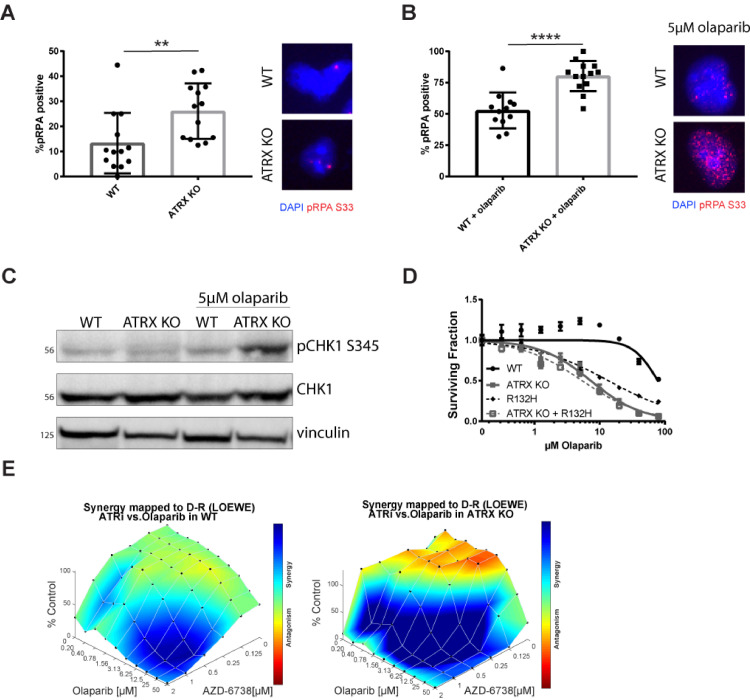
Olaparib leads to increased replication stress in ATRX KO immortalized astrocytes. (A) pRPA32 S33 foci in ATRX KO cells compared to WT. Cells with greater than 5 foci were marked positive and mean ± standard deviation of 12 fields of view plotted. Student's *T* test performed to indicate significance (*P* < 0.01). (B) pRPA32 S33 foci and (C) pCHK1 S345 western blot were performed 24 h after 5 µM olaparib treatment. For (B) Cells with greater than 10 foci were marked positive and mean ± standard deviation plotted. Student's T test performed to indicate significance (*P* < 0.0001). (D) Short term viability assay with olaparib comparing the combination of ATRX and IDH1 mutation to each mutation alone. (E) Synergy experiments were performed with olaparib and AZD-6738. Cells were treated for 96 h and the Loewe method was used to calculate synergy.

Given our findings that loss of ATRX is associated with a higher fraction of S-phase cells (Fig. 1F), and increased sensitivity to PARP inhibitors (Fig. 2), we considered the possibility that ATRX KO cells have elevated levels of replication stress. Phosphorylated RPA (pRPA) foci is a well-established marker for elevated replication stress, as RPA coats single-stranded DNA at stalled/collapsed replication forks and is phosphorylated at residue S33 in direct response to ATR activation [15,16]. Indeed, we observed elevated levels of pRPA S33 foci (Fig. 3A) in undamaged ATRX KO cells. The pRPA levels were increased after olaparib exposure, and the induction was significantly greater in ATRX KO versus WT cells (Fig. 3B). We validated this phenotype in our dox-inducible U251 shATRX cell line (Supplemental Fig. 3A). Enhanced pRPA foci after olaparib exposure correlated with phosphorylation of another ATR target, Chk1, as detected by western blot analysis in ATRX KO cells (Fig. 3C). We found that an increase in pCHK1 levels was not detected in untreated ATRX KO cells, unlike seen with pRPA levels. Since the pRPA levels were only slightly increased in these cells compared to treated cells, the amount of pCHK1 increase might not be detectable by western blot, or the levels of RPA might not be enough to induce robust ATR activation. However, in the presence of PARP inhibition, this threshold of activation is past, and increased pCHK1 levels are observed.

As described earlier, ATRX and IDH1/2 mutations co-occur frequently in glioma, and we and others have reported that the latter induce HR defects and PARP inhibitor sensitivity [23], [24], [25], [26], [27]. We thus wanted to understand how PARPi sensitivity would change in the context of IDH1/2 mutations. We engineered our immortalized astrocyte ATRX WT and KO cell line models to contain a dox-inducible IDH1-mutant (R132H) expression vector, and single cell clones were selected and validated by western blot analysis (Supplemental Fig. 4B). In these cells, the double mutant, ATRX KO/IDH1 R132H, behaved similarly to ATRX deficiency alone with olaparib treatment (Fig. 3C) as well as BMN-673 (Supplemental Fig. 4C). Additionally, we generated combination mutants in the U251 shATRX cell line (Supplemental Fig. 4D) and validated that olaparib sensitivity is equivalent in the shATRX, IDH1-mutant and double mutant models (Supplemental Fig. 4E). The findings in our immortalized astrocyte model with olaparib were validated in our U251 model by clonogenic survival assay (Supplemental Fig. 4F).

Finally, ATR and PARP inhibitor combinations have enhanced synergy in tumors with replication stress and/or HR defects [31], [32], [33], and work from our laboratory suggests that this combination is particularly effective against IDH1/2-mutant tumors [42]. Our data suggest that loss of ATRX activates the ATR signaling axis and induces replication stress. These findings prompted us to test for synergy with ATR and PARP inhibition. We observed robust synergy with this combination in our models, with maximal synergy seen in the ATRX KO cells as compared to WT (Fig. 3E). These data further highlight the dependence of ATRX KO cells on ATR signaling, and support the use of a PARP and ATR inhibitor combination against tumors with these mutations.

## Discussion

In this study, we created an isogenic pair of cell lines in immortalized astrocytes using CRISPR-Cas9-based gene editing to knockout ATRX. We investigated DNA repair efficiency through a focused drug screen of DNA damaging agents and repair inhibitors and discovered that ATRX KO cells have increased sensitivity to poly(ADP)-ribose polymerase (PARP) inhibitors compared to WT. PARP inhibitor sensitivity was due to increased replication stress as identified through increased activation of the ATR signaling pathway. In both immortalized astrocytes and glioma cells, IDH1 mutation and ATRX deficiency together lead to similar levels of PARP inhibitor sensitivity compared to the individual mutations, suggesting an epistatic interaction. Additionally, we determined that ATRX KO leads to even greater PARP and ATR inhibitor synergy than seen in wild-type cells. Overall, our data suggest that ATRX is a potential biomarker for PARP inhibitor sensitivity and DDR deficiencies, independent of IDH1/2 mutation.

Further work can be done to identify the cause of increased ATR activation, and therefore replication stress in these cells. Previous studies have shown that ATRX could be involved in replication as it can bind the replication fork complex MRE11, RAD50, NBS1 (MRN) [8], [9], [10]. The MRN complex can degrade a stalled fork and allow for replication restart, which is important for replication to continue [43]. These authors hypothesize that ATRX sequesters MRN to prevent fork degradation and decrease fork stalling [8], [9], [10]. In the absence of ATRX, an increase in proper fork degradation can lead to an overall increase in replication stress. This interaction could be the cause of olaparib sensitivity in ATRX KO cells and should be further explored in the context of PARP inhibition. There is also evidence that PARP inhibition can cause replication stress as well, so the combination of these two defects could lead to decreased ability to replicate DNA, and successfully survive [44], [45], [46].

Our work shows in two different human cell model systems, combined ATRX and IDH1 mutations, lead to a DNA repair defect, despite previous literature suggesting otherwise [30]. Interestingly, both the IDH1 and ATRX mutations lead to chromatin aberrations. IDH1 mutation leads to an increase in H3K9 trimethylation, an important signal for recruitment of DNA repair proteins [24]. ATRX is required for the deposition of H3.3, which can carry this epigenetic mark [4,47]. Further work is needed to clarify the mechanism of interplay between the two mutations, however, the double mutant (ATRX/IDH1) leads to equivalent sensitivity to olaparib when compared to either single mutation suggesting similar repair pathways are impacted.

It was also very promising to identify sensitivity to the combination of PARPi (olaparib) and ATRi (AZD 6738) in the ATRX KO cells (Fig. 3E). While both of these compounds have been used clinically, this specific combination is also currently being tested in the clinic for its anti-cancer effect [48,49]. It is currently suggested that in these dual treated cells, DNA damage due to PARPi is not properly repaired before mitosis as the cell cycle is not halted by ATR activation (due to ATRi) [31], [32], [33]. While this combination has effects in wild-type cells, it has been shown that DNA repair deficient cells have even greater sensitivity [31], [32], [33]. Our work suggests that ATRX loss leads to a DNA repair defect that can lead to this increased synergy, further widening the patient population that can benefit from this treatment regime.

As molecular classification of tumors becomes more advanced in the age of precision medicine, it is crucial to understand how specific mutations can affect treatment choices for patients. Developing efficient ways to model and characterize mutations in vitro, such as ATRX, are important steps towards achieving this goal. These models can allow for the identification of potentially synergistic targets for specific cancer types, such as ATRX loss and PARP inhibition. This combination and many more can be further explored to assist in accelerating the discovery of potential novel treatments.

## Declaration of Competing Interest

None.

## Appendix Supplementary materials

Image, application 1

Image, application 2
