# Supplementary material for: Loss of ATRX confers DNA repair defects and PARP inhibitor sensitivity
Source: Transl Oncol. 2021 Jun 9;14(9):101147. doi: 10.1016/j.tranon.2021.101147 (PMC8203843; doi:10.1016/j.tranon.2021.101147)
Supplement: Supplementary file 1 [file mmc1.docx]

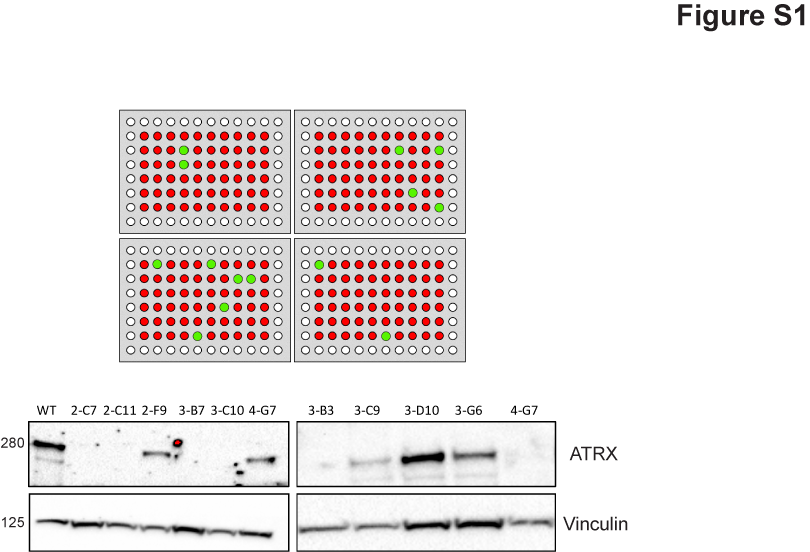


Figure S1: Utilization of immunofluorescence foci pipeline. Representation of screen for immortalized astrocyte clones. Potential hits labelled in green were expanded for further analysis. Representative western blots shown below of selected clones.


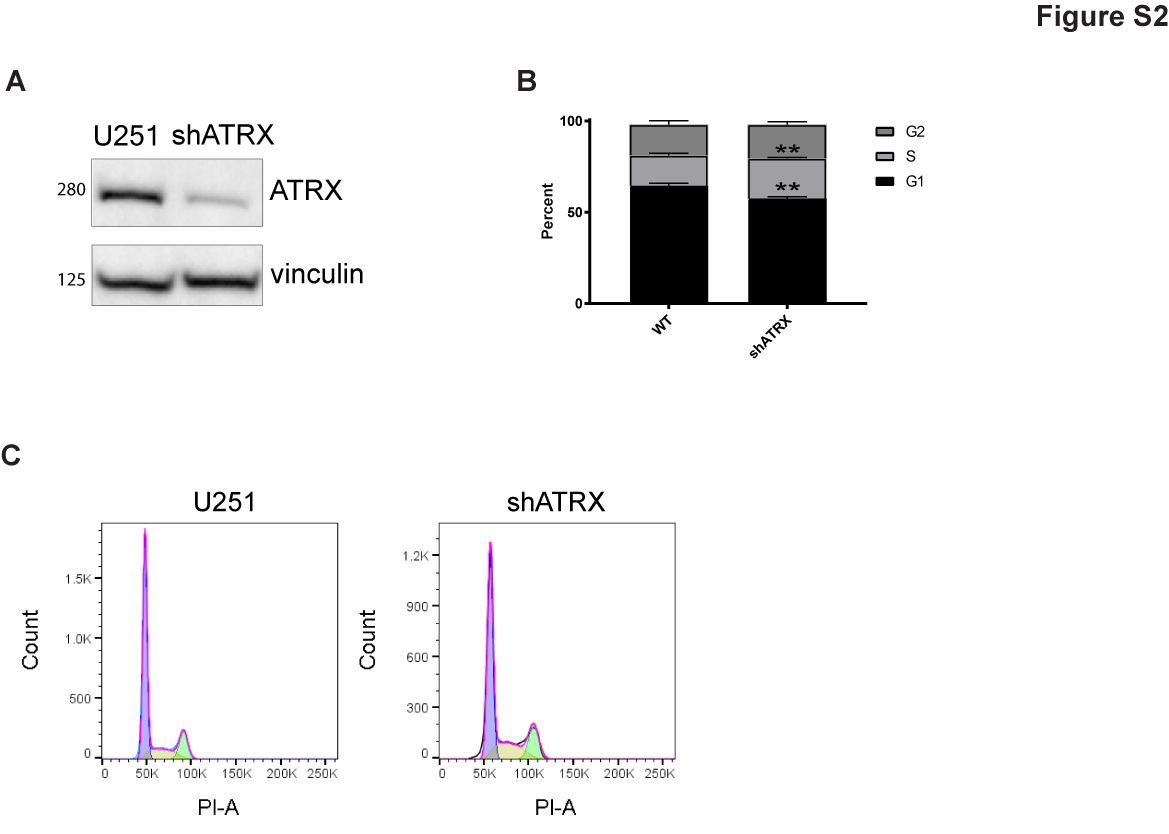


Figure S2. U251 shATRX model shows increase in S-phase population. A) Confirmation of shRNA knockdown through western blot. shRNA was induced with 1µg/ml of doxycycline for at least 96 hours before experiments. B) Percent of U251 cells in each cell cycle phase. Triplicate plotted as mean ± standard deviation. Student’s T test showed significant increase in S-phase population and decrease in G1 population (P<0.01). C) Representative propidium iodide area (PI-A) flow cytometry plots for U251 WT and shATRX cells. Purple represents G1, yellow represents S and green represents G2. Cell cycle phases were identified using the Dean-Jett-Fox model using FlowJo.

**
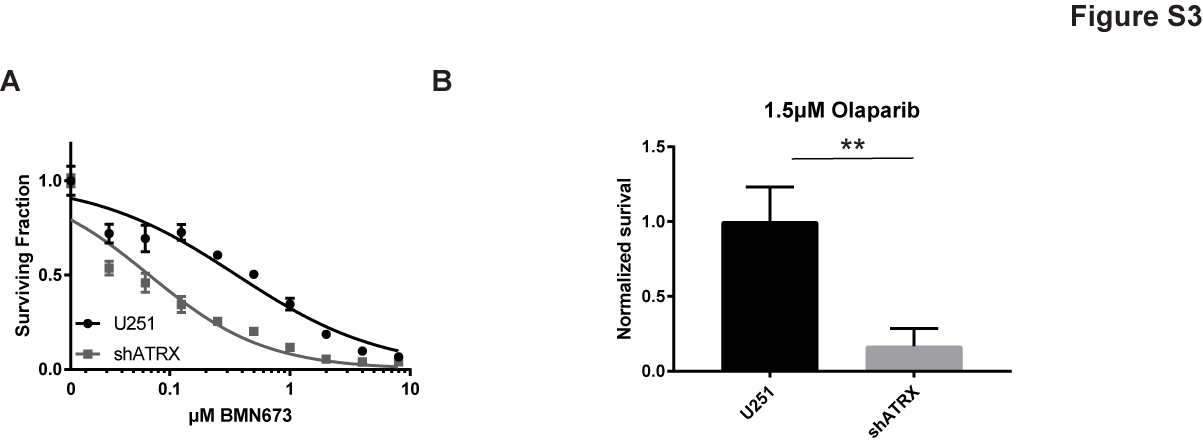
**

Figure S3: Knockdown of ATRX in U251 cells leads to PARPi sensitivity similar to astrocytes ATRX knockout. A) Short term viability assay with BMN673. B) Clonogenic survival assay with 1.5µM olaparib shows increased sensitivity in shATRX cells 14 days after treatment. Mean ± standard deviation plotted and student’s T test indicates significance (P<0.01)


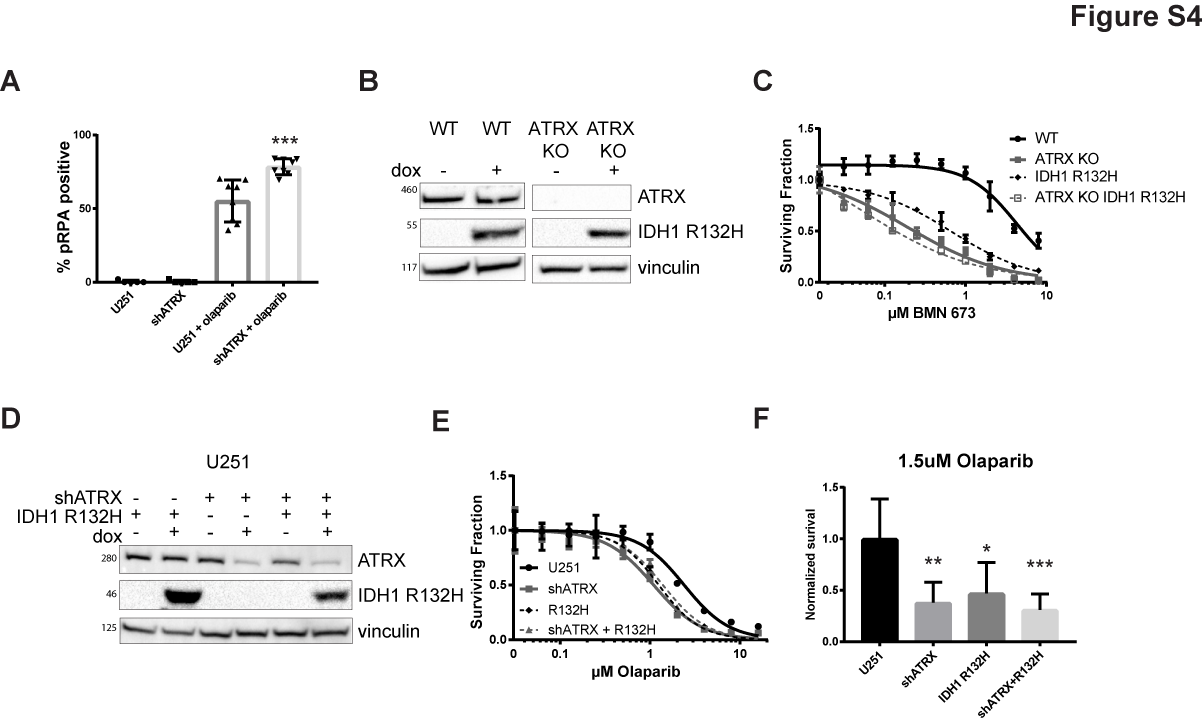
Figure S4: Olaparib leads to increased replication stress in immortalized astrocyte ATRX knockout and U251 shATRX cells. A) pRPA32 S33 foci in U251 cells. Studies were performed at 24 hours after 2 µM olaparib treatment. Cells with greater than 10 foci were marked positive. Percent of positive cells in 6 field of view were plotted. B) Western blot showing ATRX KO and IDH1 R132H over expression individually and in combination in immortalized astrocytes. C) Short term viability assay in immortalized astrocytes with BMN-673 comparing the combination of ATRX KO and IDH1 R132H mutation to each mutation alone. D) Western blot showing induction of ATRX knockdown and IDH1 R132H over expression in U251 cells. E) Short term viability assay in U251 cell lines with BMN-673. F) Clonogenic survival assay with 1.5 µM olaparib comparing all four U251 cells lines. Mean ± standard deviation plotted. Significance was calculated using a Student’s T test compared to WT. shATRX P<0.01, IDH1 R132H P<0.05, and shATRX IDH1 R132H P<0.0001. For A-F) shATRX and/or IDH1 R132H expression was induced with 1 µg/ml of doxycycline for at least 96 hours prior to experiment.
