# Supplementary material for: Loss of ATRX confers DNA repair defects and PARP inhibitor sensitivity
Source: Transl Oncol. 2021 Jun 9;14(9):101147. doi: 10.1016/j.tranon.2021.101147 (PMC8203843; doi:10.1016/j.tranon.2021.101147)
Supplement: Supplementary file 2 [file mmc2.docx]

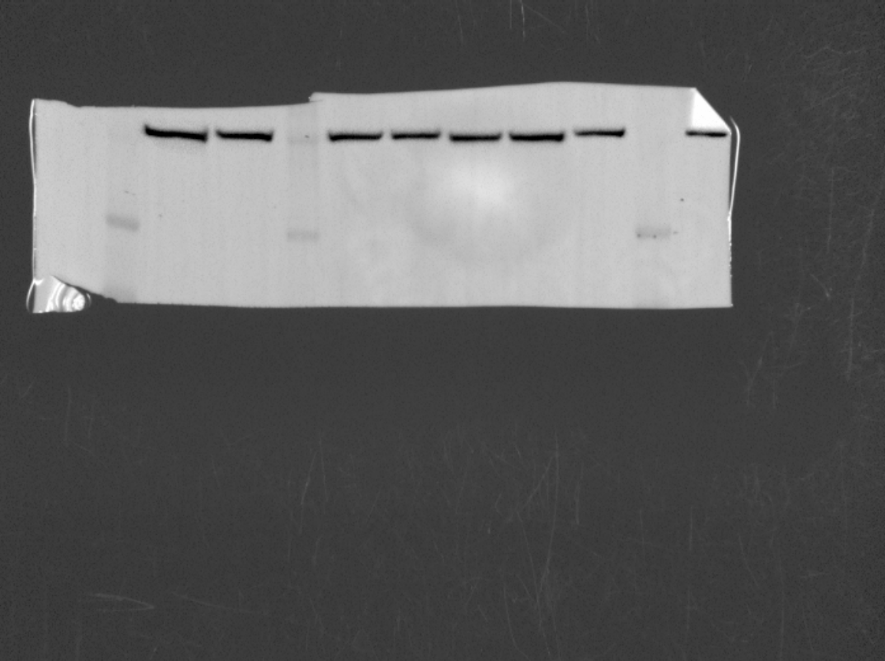

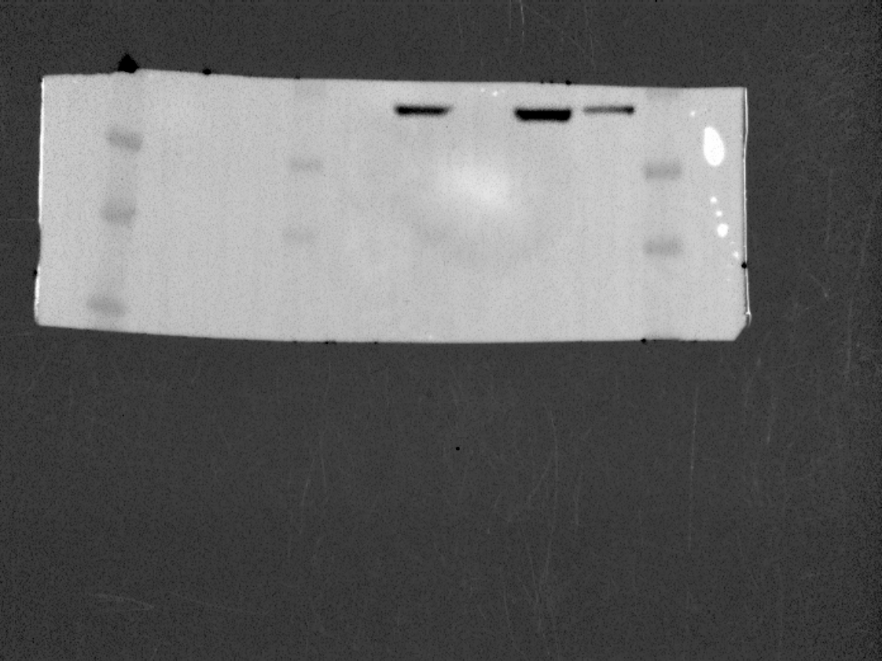

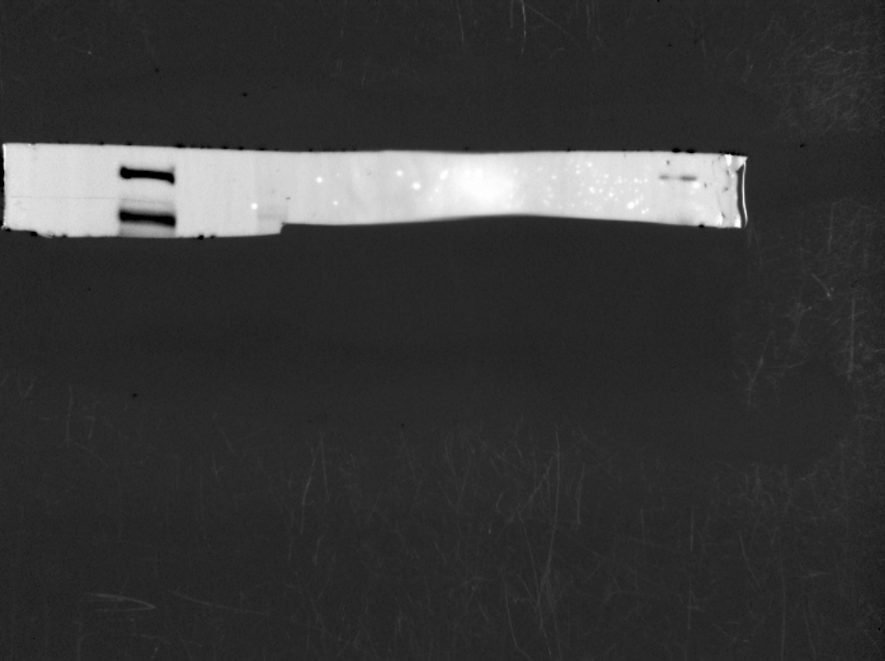
Figure 1/Figure S4:

WT

ATRX KO

ATRX KO+R132H

Dox:

+

+

WT

ATRX KO

ATRX KO+R132H

Dox:

+

+

WT

ATRX KO

ATRX KO+R132H

Dox:

+

+

IDH1 R132H

vinculin

ATRX


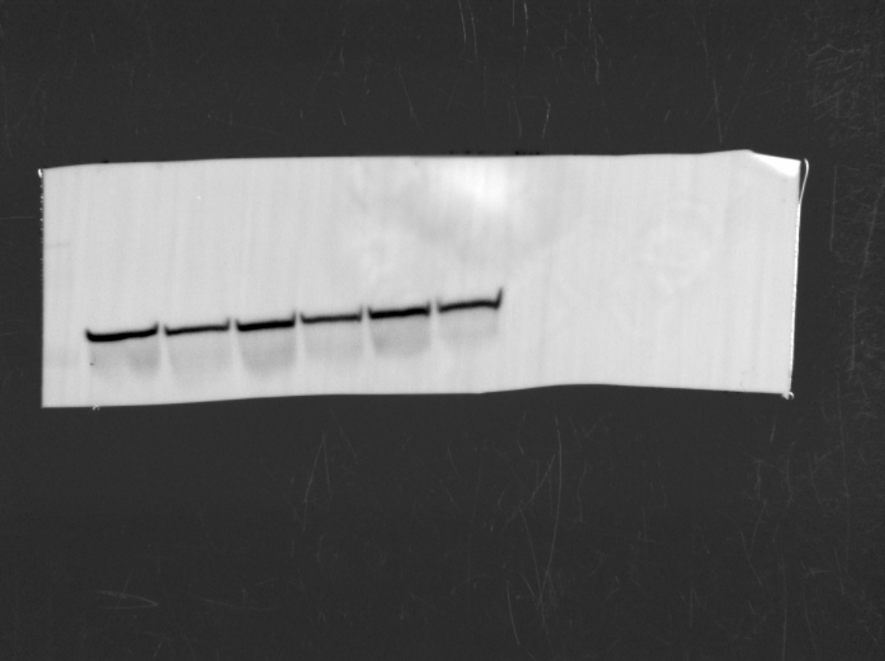

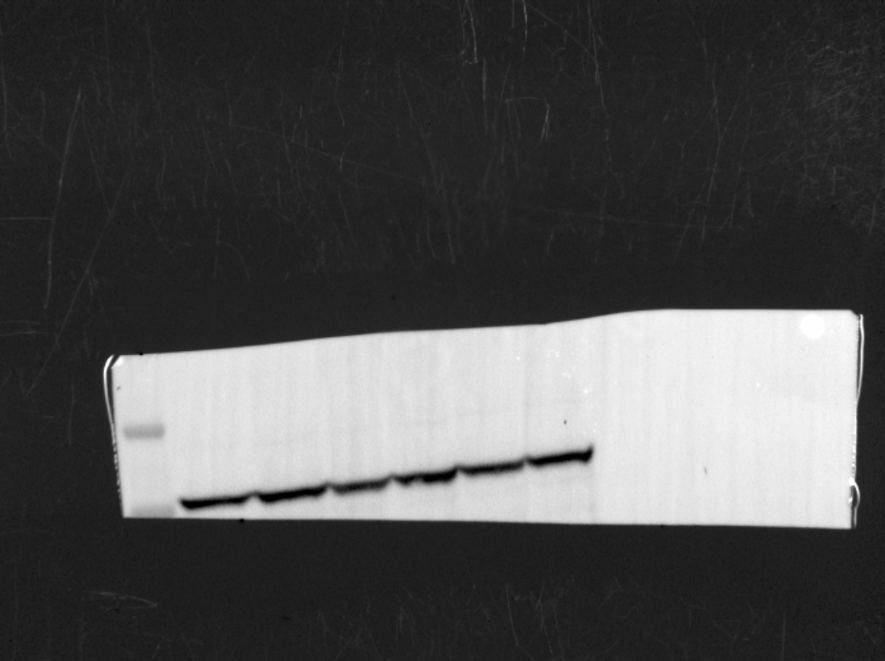

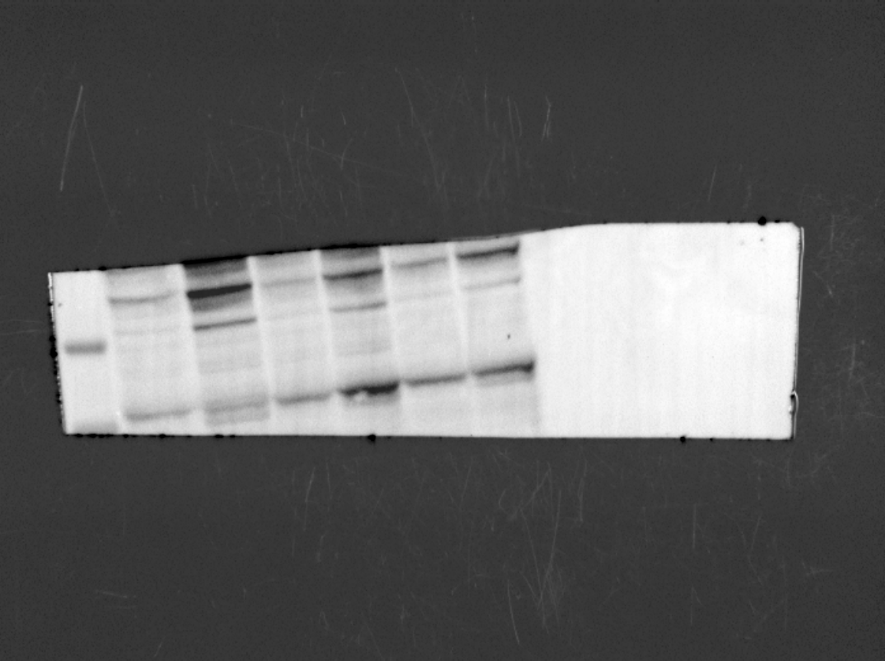
Figure 3:

olaparib

KO

WT

KO

WT

olaparib

KO

WT

KO

WT

olaparib

KO

WT

KO

WT

vinculin

CHK1

pCHK1 S345

Figure S1:


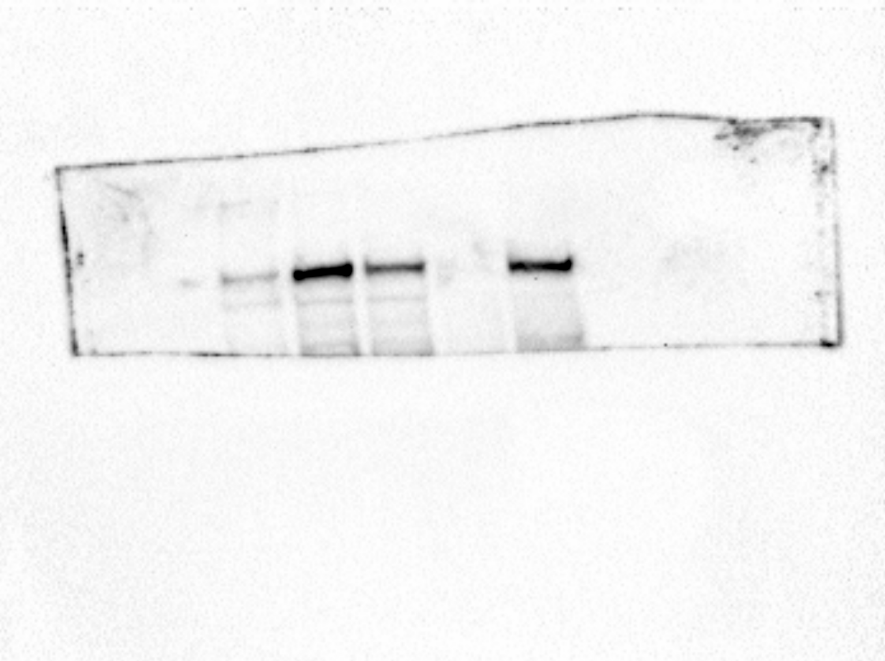

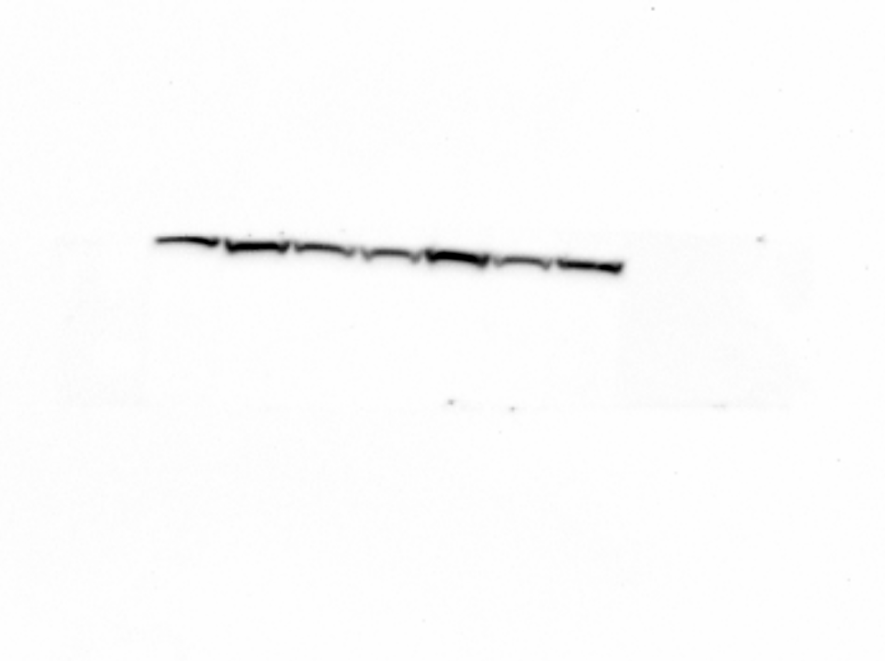

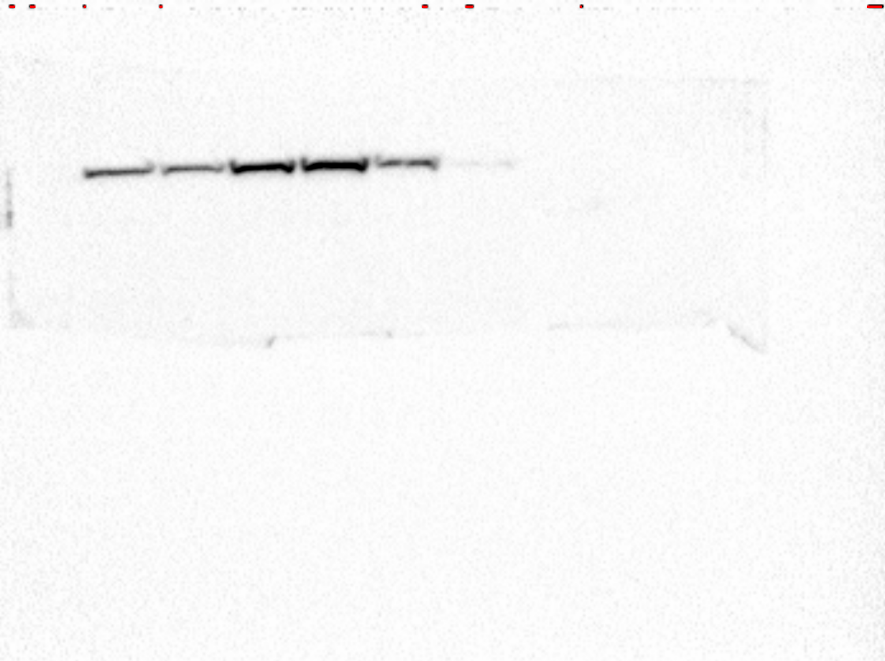

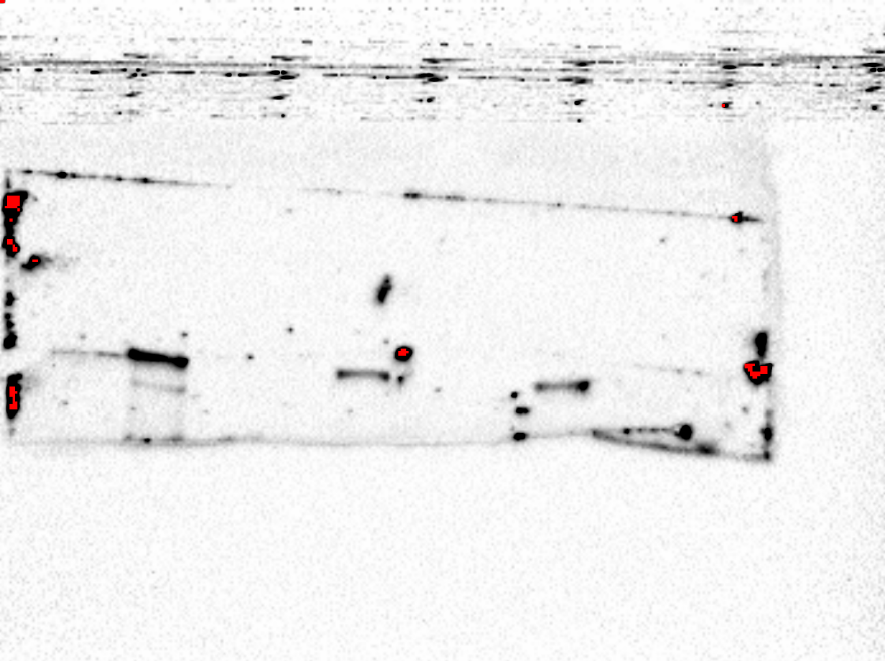


vinculin

vinculin

ATRX

ATRX


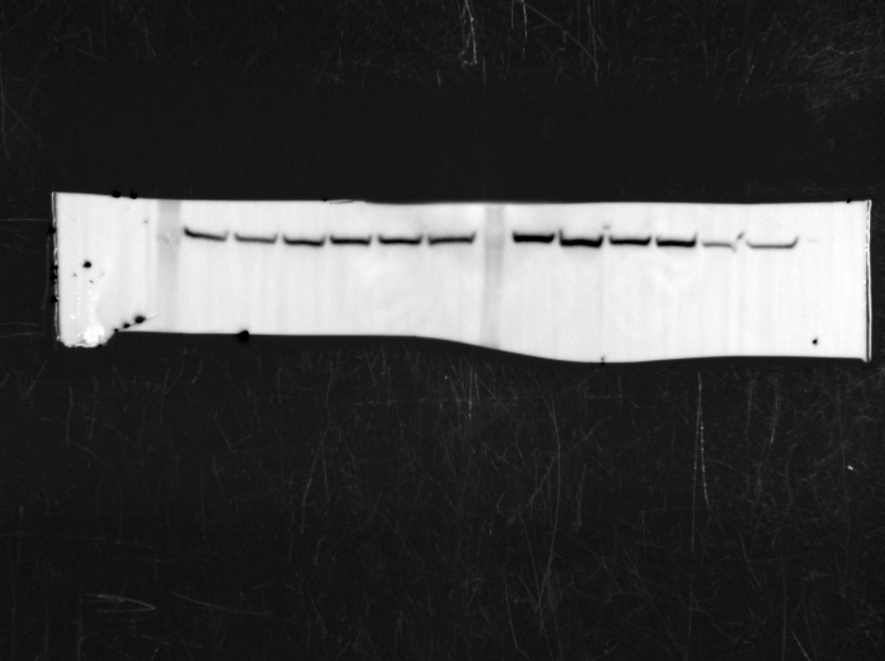

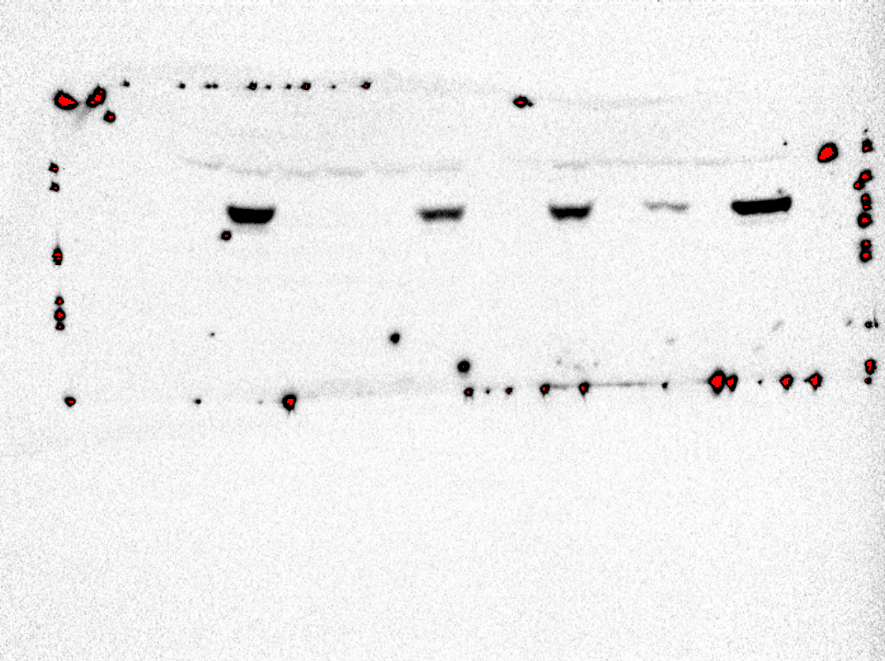

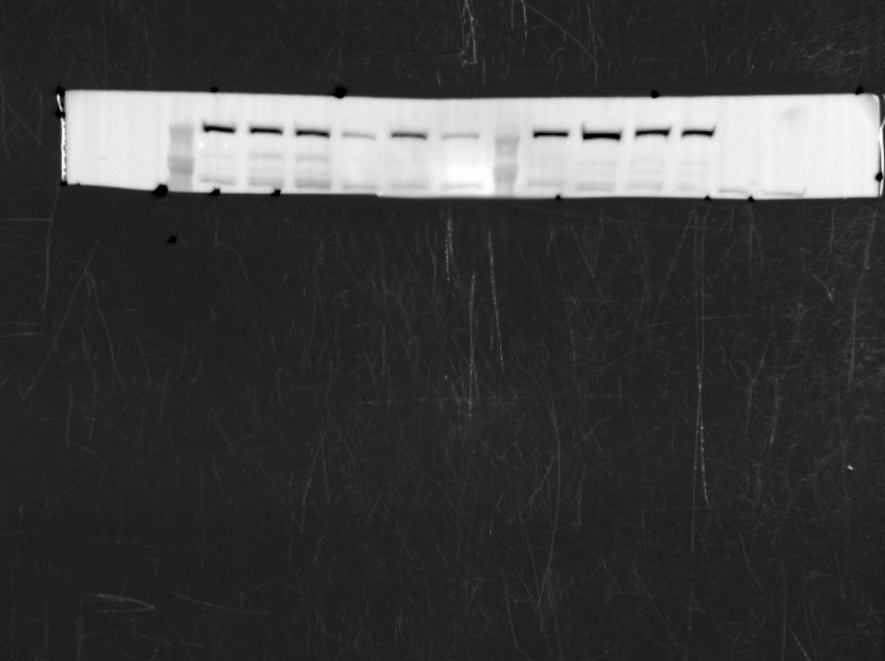
Figure S2/S4:

shATRX

R132H

shATRX

R132H

+

+

+

Dox:

shATRX

R132H

shATRX

R132H

+

+

+

Dox:

R132H

shATRX

shATRX

R132H

Dox:

+

+

+

vinculin

IDH1 R132H

ATRX


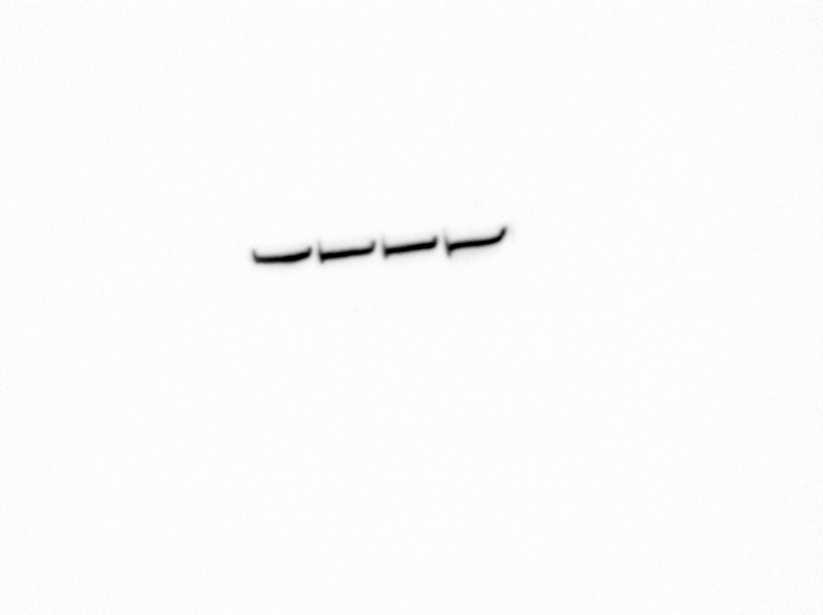

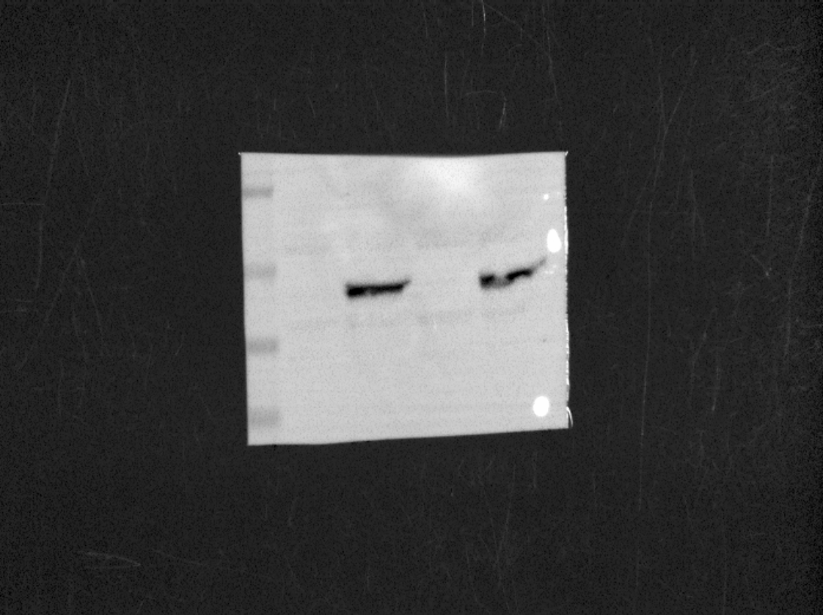

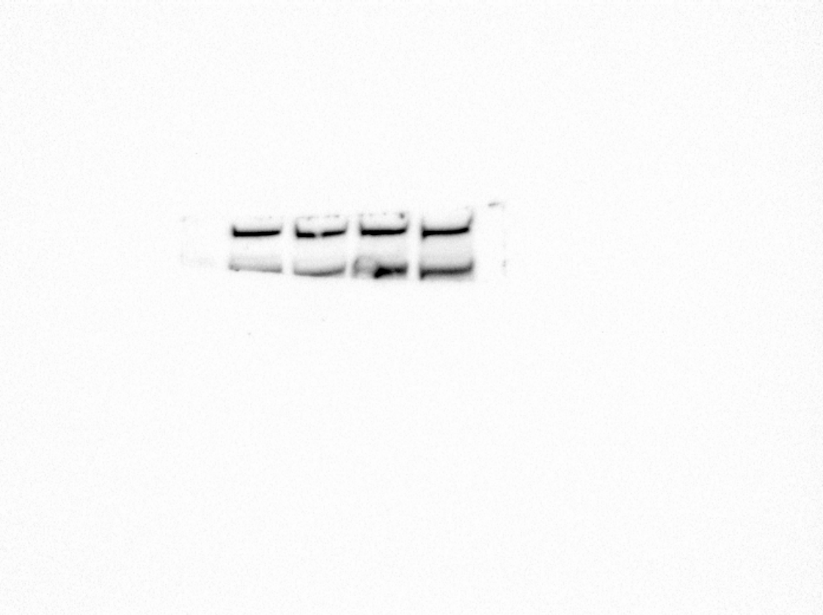
Figure S4:

Dox:

+

+

+

+

Dox:

+

+

Dox:

WT + R132H

ATRX

IDH1 R132H

vinculin
